# Molecular mechanisms of foliar water uptake in a desert tree

**DOI:** 10.1093/aobpla/plv129

**Published:** 2015-11-13

**Authors:** Xia Yan, Maoxian Zhou, Xicun Dong, Songbing Zou, Honglang Xiao, Xiao-Fei Ma

**Affiliations:** 1 Key Laboratory of Inland River Ecohydrology, Cold and Arid Regions Environmental and Engineering Research Institute, Chinese Academy of Sciences, Lanzhou 730000, PR China; 2 Key Laboratory of Stress Physiology and Ecology in Cold and Arid Regions of Gansu Province, Cold and Arid Regions Environmental and Engineering Research Institute, Chinese Academy of Sciences, Lanzhou 730000, PR China; 3 School of Agriculture and Forestry Economics and Management, Lanzhou University of Finance and Economics, Lanzhou 730020, PR China; 4 Department of Radiobiology, Institute of Modern Physics, Chinese Academy of Sciences, Lanzhou 730000, PR China

**Keywords:** Aquaporins, drought adaptation, foliar water uptake, *PIP2-1*, *Tamarix ramosissima*

## Abstract

Water deficits severely affect growth, particularly for the plants in arid and semiarid regions of the world. In addition to precipitation, other subsidiary water, such as dew, fog, clouds and small rain showers, may also be absorbed by leaves in a process known as foliar water uptake. With the severe scarcity of water in desert regions, this process is increasingly becoming a necessity. Studies have reported on physical and physiological processes of foliar water uptake. However, the molecular mechanisms remain less understood. As major channels for water regulation and transport, aquaporins (AQPs) are involved in this process. However, due to the regulatory complexity and functional diversity of AQPs, their molecular mechanism for foliar water uptake remains unclear. In this study, *Tamarix ramosissima*, a tree species widely distributed in desert regions, was investigated for gene expression patterns of AQPs and for sap flow velocity. Our results suggest that the foliar water uptake of *T. ramosissima* occurs in natural fields at night when the humidity is over a threshold of 85 %. The diurnal gene expression pattern of *AQP*s suggests that most *AQP* gene expressions display a circadian rhythm, and this could affect both photosynthesis and transpiration. At night, the *PIP2-1* gene is also upregulated with increased relative air humidity. This gene expression pattern may allow desert plants to regulate foliar water uptake to adapt to extreme drought. This study suggests a molecular basis of foliar water uptake in desert plants.

## Abstract (precis)

Foliar water uptake (FWU) plays a significant role in maintaining the stability of fragile ecosystems. However, to date, the molecular mechanism of FWU is not fully understood. Hereby, we thoroughly elucidated the relationship of sap flow velocity and the expression patterns of Aquaporin genes in a desert plant *Tamarix ramosissima*, and found that only *PIP2-1* was up-regulated with increasing relative air humidity at night. This result suggested that the *PIP2-1* gene could be in charge of regulating FWU*.* This is the first time a molecular mechanism for regulating FWU that would allow desert plants to adapt to extreme drought has been documented.

## Introduction

Water plays an important role in plant growth and ecosystem functions. Precipitation occurring as rain or snow is assumed to be the only significant source contributing to the water balance of terrestrial ecosystems (Stephenson 1990). However, to mitigate water stress, plants in arid and semiarid areas can absorb diverse sources of water through leaves (Chaves *et al*. 2002; Lioubimtseva and Henebry 2009). The sources of water include dew (Duvdevani 1964; Fritschen and Doraiswamy 1973; Kidron 2000; Zhuang and Zhao 2010; Zhuang and Ratcliffe 2012), fog (Dawson 1998; Burgess and Dawson 2004; Simonin *et al*. 2009; Berry *et al*. 2014), clouds (Pierce *et al*. 2002; Eller *et al*. 2013; Goldsmith *et al*. 2013; Berry and Smith 2014), light rain showers that only wet the leaves and high humidity (Breazeale *et al*. 1950; Fordham *et al*. 2001; Hosy *et al*. 2003; Levin *et al*. 2007; Breshears *et al*. 2008; Ewing *et al*. 2009; Arve *et al*. 2011; Kuwagata *et al*. 2012; Li *et al*. 2014). Water acquisition strategies by leaves, known as foliar water uptake (Limm *et al*. 2009), play a significant role in maintaining the stability of fragile ecosystems (Fordham *et al*. 2001; Grangeot *et al*. 2006; Hultine *et al*. 2006; Fisher *et al*. 2007; Eller *et al*. 2013). Over 70 plant species in seven different ecosystems have been documented to use foliar water uptake (Goldsmith *et al*. 2013). In the coastal prairie ecosystem of California, ∼28–66 % of water intake relies on foliar water uptake (Corbin *et al*. 2005), whereas this per cent is 34 % for the redwood tree ecosystem (Dawson 1998) and 74 % for the Israel Negev desert (Kidron 2000). Recently, with the scarcity of aggregated water in local regions, foliar water uptake is becoming increasingly important and indispensable to plants' survival.

Data suggest that water potential gradients between the soil–plant–atmosphere continuums (SPAC) drive the direction of water transport (Philip 1966). Potential scenarios for the movement of water through plants, based on water potential gradients (*ψ*), are (i) water moves from a higher *ψ*_soil_ to lower *ψ*_atmosphere_ by transpiration; (ii) water moves from a higher *ψ*_atmosphere_ (during a leaf wetting event) to a lower *ψ*_stem_ by foliar water uptake, while also simultaneously moving from a higher *ψ*_soil_ to a lower *ψ*_stem_, thus refilling the plant from two directions and (iii) water moves from a higher *ψ*_atmosphere_ (during a leaf wetting event) to a lower *ψ*_soil_ by foliar water uptake. Once an optimal water potential gradient is established, the bidirectional movement of water through the stomata does not stop (Eller *et al*. 2013). The traditional scenario, in which water moves unidirectionally from the soil, through a plant and into the surrounding atmosphere, has been well studied, whereas less is known about the other two scenarios. Techniques such as sap flow analyses, use of stable isotopes, apoplastic tracers and ecophysiological measurements under field and greenhouse conditions have been used to study the physical and physiological characteristics of plants (Eller *et al*. 2013, 2015). However, the intrinsic response, including the gene expression pattern, has not been fully investigated to understand the molecular mechanism of foliar water uptake (Ohrui *et al*. 2007; Laur and Hacke 2014).

Aquaporins (AQPs) have been documented as major channels for controlling water content within plants by regulating the short-term stomata movement (Chaumont *et al*. 2001; Burkhardt 2010; Burkhardt *et al*. 2012; Chaumont and Tyerman 2014; Nada and Abogadallah 2014). The differential expression of AQPs is believed to direct water transport (Levin *et al*. 2007, 2009; Vandeleur *et al*. 2009; Šurbanovski *et al*. 2013), and has been reported to regulate foliar water uptake in two different plant species (Ohrui *et al*. 2007; Laur and Hacke 2014). There are five major subfamilies of AQPs: plasma membrane intrinsic proteins (PIPs), tonoplast intrinsic proteins (TIPs), noduline-26-like intrinsic proteins (NIPs), small basic intrinsic proteins and uncharacterized intrinsic proteins (Chaumont *et al*. 2001). The subfamily of proteins displays a high diversity of localization and functional specialization in plant cells (Maurel *et al*. 2008; Wudick *et al*. 2009). Even homologous genes have different expression patterns due to the different hydraulic conductivity among plant species [e.g. *PIP1* is unimodally expressed in the roots of *Lotus japonicus* (Hachez *et al*. 2008) and is bimodally expressed in *Vitis vinifera* (Moshelion *et al*. 2002)]. The diversity of functions and multiple molecular regulations of AQPs have been well studied in model plants such as *Arabidopsis* (Sade *et al*. 2010, 2014; Heckwolf *et al*. 2011; Eisenach *et al*. 2012; Xin *et al*. 2014), rice (Hanba *et al*. 2004; Lian *et al*. 2004; Sakurai *et al*. 2005; Kawase *et al*. 2013; Yooyongwech *et al*. 2013; Nada and Abogadallah 2014) and maize (Bastías *et al*. 2004; Hachez *et al*. 2008; Heinen *et al*. 2014). However, it is less studied in the non-model plants among different ecosystems (Chaumont and Tyerman 2014; Heinen *et al*. 2014; Laur and Hacke 2014; Nada and Abogadallah 2014; Perez-Martin *et al*. 2014). *Tamarix ramosissima*, a perennial desert forest tree species, is distributed in desert regions in northern China with annual precipitation under 200 mm (Yu *et al*. 2013). It plays a significant role in sand fixation and soil and water conservation. A previous study demonstrated that foliar water uptake may be an important strategy for *T. ramosissima* to mitigate drought when high atmospheric humidity occurs (Li *et al*. 2014). The radial flow of water across the roots could be reflected by changes in sap flow velocity and other physiological parameters. However, how the water potential gradient is built for solute accumulation and how water storage occurs in cells and plants are not clear.

In this study, we combined the monitoring of sap flow velocity and meteorological factors in the field with reverse transcription polymerase chain reaction (RT-PCR) to quantify the transcription patterns of *AQP*s during a clear cloudless day, to determine the major genes responsible for the process of foliar water uptake and to identify molecular mechanisms of the foliar water uptake of *T. ramosissima*. This study will help to determine, at the molecular level, how desert plants use limited water resources by absorbing air moisture from leaves.

## Methods

### Site description

Two-year-old *T. ramosissima* seedlings were transplanted in 2003 in a uniform matrix by spaced, 2 m columns and 4 m rows in an ecological station located in Jingtai County (104.07E, 37.15N) at the southern edge of the Tenger Desert. None of the plants were watered since transplantation. During the year 2013, the amount of sunshine was 2726 h with >60 % days of sunshine. The annual radiation dose was 147.8 kcal cm^−1^. The largest amount of precipitation occurred from June to September, and each precipitation event was below 3 mm (maximum 3 mm on 26 July). Precipitation below 0.2 mm occurred 70 times for a total of 14 mm, precipitation ranging from 0.4 to 0.8 mm occurred 66 times for a total of 34.4 mm and precipitation from 1 to 3 mm occurred 23 times for a total of 37 mm. Apparently, these short intervals of rain showers could only wet the plant leaves but did not significantly contribute to absorption by the roots. Due to the small amount of rain, the diurnal soil water content at a depth of 20 cm was low and varied slightly at ∼3.79 % in the morning, 3.60 % at noon and 3.80 % at predawn **[see** **Supporting Information—File S1****]**. Soil water potential also varied slightly along the soil depth **[see Supporting Information—Fig. S1]**. The contribution of air moisture to the plants cannot be ignored for plants in this arid area. The average relative humidity (RH) was 47 % from June to September, the lowest was ∼8 % in the middle of a sunny day in June and the highest was over 95 %, which condensed into dew at night. The diurnal variation in the relative air humidity (AirRh) from June to September is shown in **Supporting Information—Fig. S2**. The mean annual temperature was 10.6 °C, and annual accumulated temperature climbed to 3614.8 °C above 0 °C, and 3038 °C above 10 °C.

### Meteorological data measurement

A standard automatic weather station (AWS Type WS01; Delta-T, Cambridge, UK) was set up ∼2 m above the soil surface to monitor meteorological factors such as wind speed (m s^−1^), rainfall (mm), AirRh (%), air temperature (AirTm,°C), soil temperature (SoilTm,°C), atmospheric pressure (hPa) and photosynthetically active radiation (PAR, mmol). The data were automatically recorded for each minute, and the mean values of each measurement were then estimated based on the average for each half-hour time period. The field experiments were conducted from 25 to 28 June 2013. It was sunny in the daytime, with clear skies at night, and there was a slight breeze.

### Sap flow velocity measurement using the heat-pulse technique

Heat-pulse sap flow sensors (SF100; Greenspan Technology, Coffs Harbour, Australia) on sap flow gauges (Flow32; Dynamax Inc., Houston, TX, USA) were used to continuously monitor sap velocity using the energy balance principle. Heat-pulse velocity was then converted into sap flow velocity using the algorithms developed by Edwards and Warwick (1984). All of the calculations were integrated using SAPCAL software. Details for the installation of the gauges were described by Li *et al*. (2014). In the diurnal experiments, three trees were selected to monitor the sap flow velocity in *in situ* positions of two roots and three stems. Each tree was wrapped with two gauges at a distance of <20 cm to obtain the integral data and eliminate the diameter effect. Singular data were manually released with comparison of two data points from the two gauges during the measurement period. However, for intermittent data occurring in one tree, two other trees were used to fit the pattern of the diurnal sap flow velocity. Therefore, a total of four gauges were used for one point in the sap flow velocity curve of the *in situ* position at 50 cm (MS), 20 cm (S20) and 10 cm (S10), and the roots were ∼50 cm (MR) and 20 cm (R20). Positive values for sap flow velocity were regarded as water storage depletion, while negative values were regarded as water replenishment or storage (Goldstein *et al*. 1998; Li *et al*. 2014), which signifies the occurrence of foliar water uptake.

### High air humidity exposure experiment

In the high moisture exposure experiment, it was hard to find plants in the natural field similar to those in the controlled cabins. Soil elements and root bifurcation could also complicate the analysis of water conduction in adult plants in field experiments. To eliminate these factors and achieve consistency, we attempted to find one tree roughly divided into two main branches, separate from the ground, with a crown width of ∼2.95 ± 0.05 m in the west–east direction and ∼3.25 ± 0.05 m in the north–south direction. One branch was exposed to natural conditions as a control, and the other was exposed to a controlled humidity chamber as treatment. We created a Plexiglas^®^ room ∼3 × 1.8 × 1.8 m that completely covered the two branches. The bottom of the Plexiglas^®^ room was completely covered with soil sealing. The joints between the Plexiglas^®^ were sealed with multilayer tape. Special doors were made in the Plexiglas^®^ room. To eliminate the effects of air convection from the open door for leaf collection, we entered the Plexiglas^®^ room 20 min before taking measurements. Two ultrasonic humidifiers (Yadu Electronics, Beijing, China) were used to increase the atmospheric moisture in the chamber. Air temperature and RH were monitored with a thermo-hygrograph (MicroLog PRO-EC750; Fourier Systems Ltd, Rosh Ha'ayin, Israel), and the values were calibrated with an automatic weather station (AWS TypeWS01; Delta-T). With the exception of humidity, the meteorological conditions were the same between the inside and outside of the glass chamber. The details of the equipment are found in Li *et al*. (2014).

### Collection of leaves for gene expression studies

To quantify the diurnal expression level of AQPs, at least 5 g of fresh leaves were collected from at least three trees for RNA extraction at 2-h intervals from 6:00 to 22:00 on 25 June 2013. Leaves were also collected from the plants in the high air humidity exposure experiment. Samples from inside branches were marked as ‘inwet-nocut’, whereas samples from outside branches were marked ‘outnowet-nocut.’ To exclude the effects of water from root absorption, two small branches were cut from the main stem of the plant and hung outside (samples marked ‘outnowet-cut’) and inside a hermetic glass house (samples marked ‘inwet-cut’). Fresh leaves were picked from at least three branches inside and outside the enclosure.

All of the leaves were placed into liquid nitrogen immediately after being collected from the tree, and were taken back to the laboratory and stored at −80 °C. Leaves from three branches as biological replicates were used to extract total RNA samples, and were then equally mixed to perform RT-PCR.

### Total RNA isolation and cDNA synthesis

Total RNA was extracted using the E.Z.N.A.^®^ Plant RNA kit (Omega Bio-Tek, Norcross, GA, USA) with 2 % polyvinylpolypyrrolidone addition, and the remaining DNA was removed with RNase-free DNase (Omega Bio-Tek) according to the manufacture's instruction manual. The total RNA concentration and purity were assessed by OD 260/280 nm ratios as determined using NanoDrop1000 (Thermo Scientific, Waltham, MA, USA), and all samples passed quality control analyses with OD 260/280 ratios ranging between 1.9 and 2.2 and OD 260/230 ratios <2.0. RNA integrity was verified by 1.5 % agarose gel electrophoresis with two distinct 28S/18S ribosomal RNA bands. For each sample, 1 µg of total RNA were reverse transcribed in a 20-µL reaction volume with oligo dT primers, using the RevertAid™ First Strand cDNA Synthesis Kit (Fermentas/Thermo Scientific). The cDNAs were diluted 1 : 10 with nuclease-free water prior to quantitative PCR analyses.

### Selection and validation of candidate genes

A total of six homologous *AQP* genes were selected based on transcriptome data from *Tamarix hispida*, with each sequence length over 900 bp and annotation *E*-values <1e−14 in the National Center for Biotechnology Information non-redundant protein database. Alignments of multiple cDNA sequences were performed using ClustalW, which was integrated using the BioEdit 7.2.5 software (Hall 1999) to exclude identical sequences. Then, the sequences were inputted into the GENSCAN Web Server (http://genes.mit.edu/GENSCAN.html) to predict peptide sequences. To verify the AQP domains, BLAST searches were performed on the orthologous protein sequences from *Arabidopsis*. Alignments of the AQP protein sequences were performed using ClustalW, which was integrated into the BioEdit 7.2.5 software (Hall 1999). A phylogenetic tree of these genes was constructed using MEGA 6 (Tamura *et al*. 2013) with the maximum likelihood method. The detailed information of the six *AQP* homologues is listed in Table 1. The protein sequences of the AQPs used for construction of the phylogenetic tree are listed in **Supporting Information—Fig. S3**.

**Table 1.** Reverse transcription polymerase chain reaction primer sequences and annotation information of *AQP* genes.

Gene ID	Length (bp)	Forward primers (5′–3′)	Reverse primers (5′–3′)	Gene name	Annotation
Unigene41312	1225	GAGTCAGTGCCATTGAAGCA	ACCAACAACGAGGCCAATAG	*TIP1-1*	AQP TIP1-1 [*Arabidopsis thaliana*]
Unigene13871	920	TCCGAAGAAGGGTAACATCG	GCTGGACCAAAAGAGACAGC	*TIP1-3*	AQP TIP1-3 [*Arabidopsis thaliana*]
Unigene15471	1314	CACCGCTGTCGCTACAGATA	CCTCATACGACGTGGAGGTT	*NIP5-1*	Putative AQP NIP5-1 [*Arabidopsis thaliana*]
Unigene5339	974	GCATAAACCTGGAGCTGGAA	TGGTGGCCCGTTTTACTAAG	*NIP7*	60S ribosome subunit biogenesis protein NIP7 homologue [*Vitis vinifera*]
Unigene23675	1261	GCGGTTACATTCGGTCTGTT	CGACAATCTCAGCACCAAGA	*PIP1-2*	AQP PIP1-2 [*Arabidopsis thaliana*]
Unigene38680	1333	ATGGTGTCCCAGTGCTTAGG	CCAAGTGAACCATGAACACG	*PIP2-1*	AQP PIP2-1 [*Arabidopsis thaliana*]
Unigene47261	2049	CCCTGTTGTTACGGAGTCGT	GATGGATCCCTTCTTCGACA	*GAPDH*	Glyceraldehyde-3-phosphate dehydrogenase

### Quantitative real-time RT-PCR

Based on the sequences of each gene from the *de novo* transcriptome of *T. hispida* (Wang *et al*. 2014), RT-PCR primers were designed using OligoPerfect Designer (http://tools.invitrogen.com/content.cfm?pageid=9716&icid=fr-oligo-6) with lengths of ∼20 bp, and the optimal melting temperatures were 60 °C with an optimal GC content of 50 %. Product sizes were between 100 and 300 bp (Table 1). Polymerase chain reaction efficiencies per sample were calculated using LinRegPCR, and all of the mean PCR efficiencies per amplicon were between 1.8 and 2.0.

A 20-µL reaction volume was used for ampliﬁcation. Each reaction contained 10 µL of DyNAmo Flash SYBR Green qPCR Kit Master Mix (Thermo Scientific), 0.5 µL of forward and reverse primers, 0.2 µL of F-402 buffer and 2 µL of cDNA synthesized from total RNA. The PCR programme contained an initial denaturation step of 5 min at 95 °C, followed by denaturation for 15 s at 95 °C, annealing for 30 s at 60 °C and extension for 30 s at 72 °C for 40 cycles. Real-time PCR was used to obtain the relative expression level of each sample using the qTOWER 2.0/2.2 thermal cycler (Analytik, Jena, Germany). Dissociation curves were obtained by heating amplicons from 60 to 95 °C **[see Supporting Information—Fig. S4]**, and each primer had good specificity, quality, efficiency and dissociation curves. The Cq value for each sample and fluorescence threshold set was determined using LinRegPCR. *GAPDH* was used as a reference gene to normalize gene expression levels, and relative quantification was determined using the ΔΔCt method (Livak and Schmittgen 2001).

### Statistical analysis

The correlations between the meteorological factors including AirRH, AirTm, SoilTm, air pressure (AirP), wind and biological responses, including sap flow velocity (in the stems and roots) and gene expression level of *AQP*s, were analysed with the Pearson correlation coefficient (PCC) using SPSS software version 17.0 for Windows (SPSS Inc., Chicago, IL, USA). The regression analyses between PAR and sap flow velocity were also performed using SPSS software.

## Results

### Diurnal variation of meteorological factors on sunny days at the field experiment site

**Figure 1.**
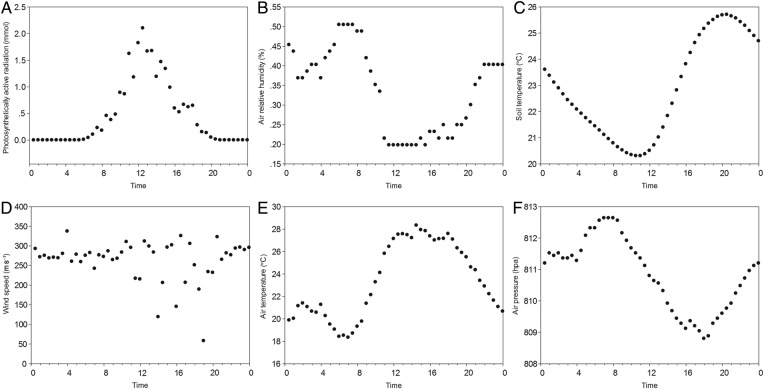
Diurnal variation in meteorological factors at the field experiment site on 25 June 2013. The diurnal change in (A) PAR (mmol), (B) AirRh (%), (C) SoilTm (°C), (D) wind speed (m s^−1^), (E) AirTm (°C) and (F) AirP (hPa).

Field work was conducted from 24 to 27 June, and we extracted all of the meteorological factors on 25 June 2013, as the weather was clear with maximal PAR of ∼2.10 mmol. Major meteorological factors related to air/water were plotted at half-hour time intervals (Fig. 1). As illustrated in Fig. 1, PAR produced a unimodal curve, which remained lowest during the day until 8:00, rose sharply and peaked at 12:30, and steadily decreased throughout the day. The patterns of other meteorological factors, such as AirRh (%) and AirP (hPa), were in the same unimodal pattern as PAR (Fig. 1B, C, E and F), but were several hours delayed because the thermal response lagged. Analysis from PCC showed that these three meteorological factors were related to PAR. We have listed only PCC between the AirRh and PAR in Table 2 (*r* = −0.875, *P* < 0.001). We ignored erratic factors, such as wind speed, in the following discussion (Fig. 1D).

**Table 2.** Correlations between meteorological factors and sap flow velocity from time 20 to 24. *Correlation is significant at the 0.05 level (two tailed). **Correlation is significant at the 0.01 level (two tailed). PCC, Pearson correlation coefficient; Sig._2, two-tailed test.

	PAR	AirRh	MS	S20	S10	MR	R20
PAR							
PCC	1	−0.875**	0.962**	0.649	0.618	0.867**	0.886**
Sig._2		0.002	0.000	0.058	0.076	0.002	0.001
AirRh							
PCC	−0.875**	1	−0.904**	−0.793*	−0.878**	−0.984**	−0.942**
Sig._2	0.002		0.001	0.011	0.002	0.000	0.000
MS							
PCC	0.962**	−0.904**	1	0.794*	0.772*	0.931**	0.943**
Sig._2	0.000	0.001		0.011	0.015	0.000	0.000
S20							
PCC	0.649	−0.793*	0.794*	1	0.880**	0.859**	0.866**
Sig._2	0.058	0.011	0.011		0.002	0.003	0.003
S10							
PCC	0.618	−0.878**	0.772*	0.880**	1	0.914**	0.844**
Sig._2	0.076	0.002	0.015	0.002		0.001	0.004
MR							
PCC	0.867**	−0.984**	0.931**	0.859**	0.914**	1	0.961**
Sig._2	0.002	0.000	0.000	0.003	0.001		0.000
R20							
PCC	0.886**	−0.942**	0.943**	0.866**	0.844**	0.961**	1
Sig._2	0.001	0.000	0.000	0.003	0.004	0.000

### Meteorological factors affect the diurnal variation of sap velocity at different positions in the sapwood of *T. ramosissima*

**Figure 2.**
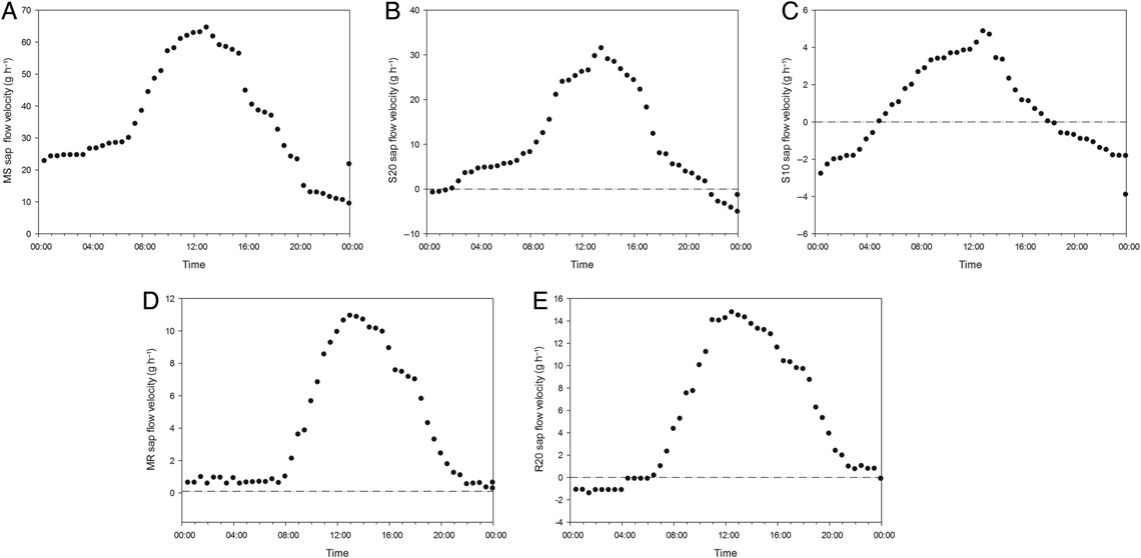
Diurnal variation in sap velocity at different positions of the sapwood of *T. ramosissima*. (A) For the main stem with a diameter of ∼30 cm (MS), (B) for the stem with a diameter of ∼20 cm (S20), (C) for the stem with a diameter of ∼10 cm (S10), (D) for the main root with a diameter of ∼30 cm (MR) and (E) for the root with a diameter of ∼20 cm (R20). The null sap flow velocity is marked with a dashed dotted line, which was used as a transition criterion to determine whether foliar water uptake occurred.

**Figure 3.**
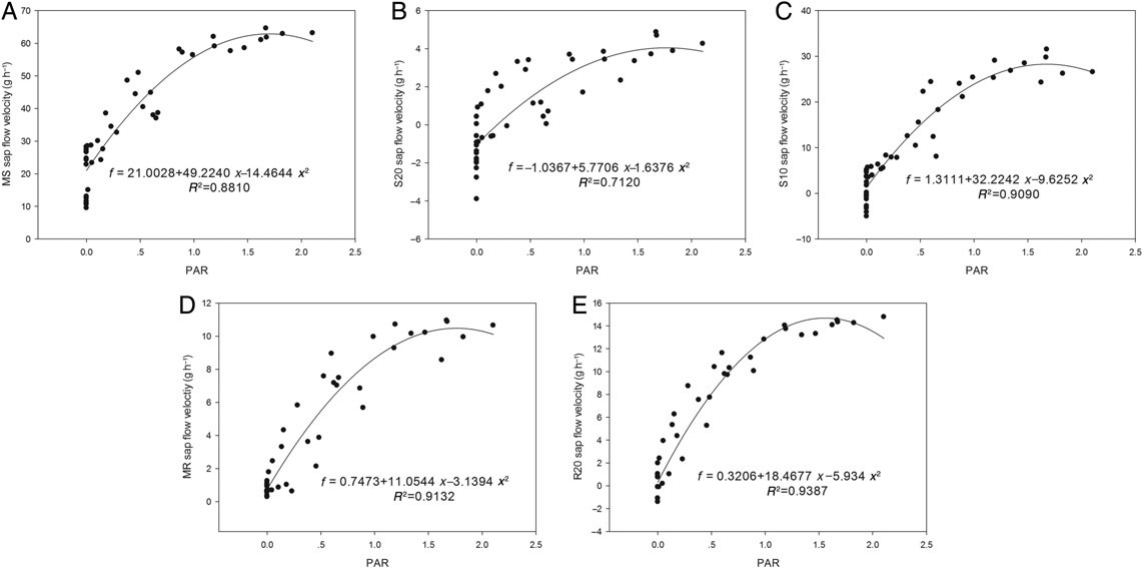
Correlation between sap flow velocity and PAR simulated by two-order line regression for each curve with 95 % CI as generated sigma plot 12.0. The scatter dot plot shows observed data from the field experiment site. The curve represented fit curve with the significant equation. (A) For the main stem with a diameter of ∼30 cm (MS), (B) for the stem with a diameter of ∼20 cm (S20), (C) for the stem with a diameter of ∼10 cm (S10), (D) for the main root with a diameter of ∼30 cm (MR) and (E) for the root with a diameter of ∼20 cm (R20).

**Figure 4.**
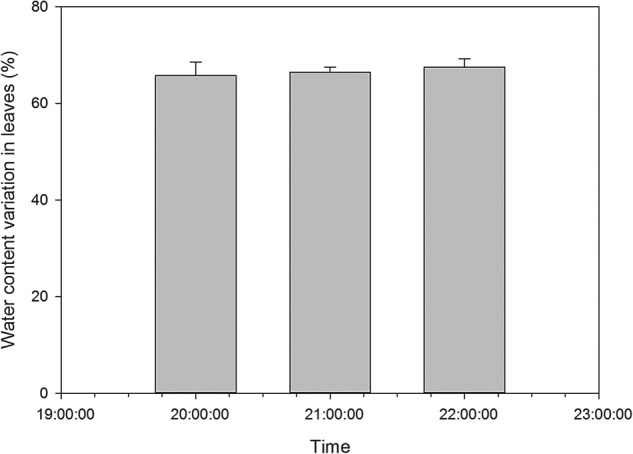
Water content in leaves and small stems from 20:00 to 22:00 on 25 June 2013.

**Figure 5.**
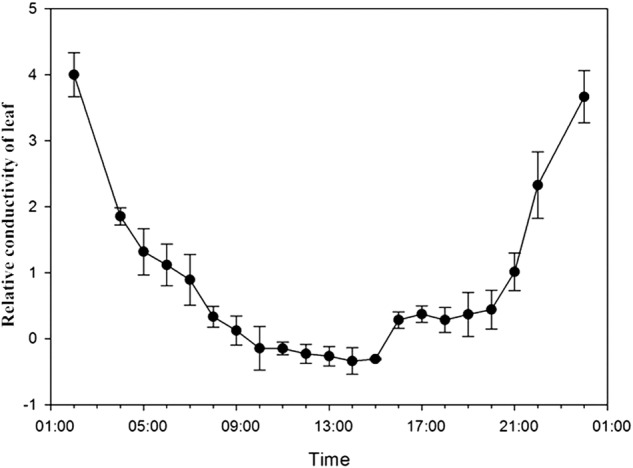
Diurnal relative conductivity of leaves on 25 June 2013.

**Figure 6.**
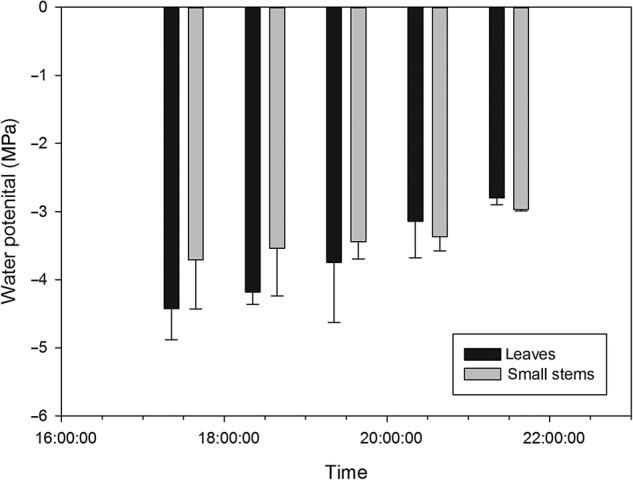
Water gradient potential in leaves and stems in *Tamarix* from 16:00 to 22:00 on 25 June 2013.

We monitored sap flow velocities at different positions in the root (MR, R20) and stem (MS, S20, S10) with an interval of 6 min on 25 June 2013. Based on meteorological data, the averages of all data within 1 half-hour were plotted (Fig. 2A–E). Sap flow velocity was initiated at 8:00, peaked at ∼12:00, remained high with slight variations for ∼4 h and decreased thereafter. The sap flow velocity continued to decrease after 20:00. The pattern of sap flow velocity had the same unimodal curve as PAR for *T. ramosissima*. The relationship between sap flow velocity and PAR fitted a quadratic regression equation *y* = *y*_0_ + *ax* + *bx*^2^ with *R*^2^ over 0.7 and *P*-value >0.0001 (Fig. 3A–E), which suggested that the sap flow velocity was greatly affected by PAR. In addition, as the weather on 25 June 2013 was clear and sunny, the diurnal soil water content was regarded to be constant, whereas the AirRh increased from 20:00, which may contribute to sap flow velocity. The water content of leaves between 20:00 and 22:00 increased with increasing AirRh (Fig. 4), and the leaf relative conductivity increased (Fig. 5). The water potential in the leaves and stems increased, and was higher in leaves than in stems from 20:00 to 22:00 (Fig. 6). Interestingly, there was a negative value for the sap flow velocity that occurred in the thinner stem after 20:00, and it varied between stems with different diameters. Thinner stems in the trunk stems had less negative values for sap flow velocity (negative value of sap flow velocity was designated as MS < S20 < S10). Accordingly, the negative PCC between the thinner stems and AirRh was greater than for those between the big stems and AirRh (Table 2). The negative sap flow velocity could represent water replenishment from foliar water uptake. These phenomena suggested that increasing AirRh changed the water potential gradient between the air and the leaves, and the leaves started absorbing air moisture, resulting in the sap reflux.

### Diurnal hydraulic adhesion of *AQP* expression in response to desert meteorological factors

**Figure 7.**
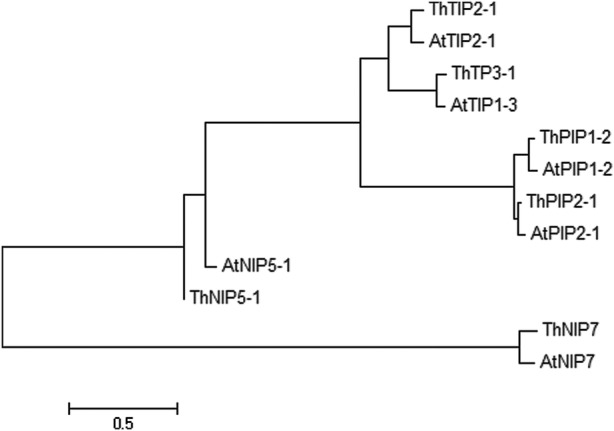
Phylogenetic relationship of the six AQPs in the *T. ramosissima*. The phylogenetic tree was constructed with maximum likelihood using the Jones–Taylor–Thornton (JTT) model in MEGA 6.0. At, *Arabidopsis thaliana*; Th, *T. ramosissima*. The distance scale is 0.5.

**Figure 8.**
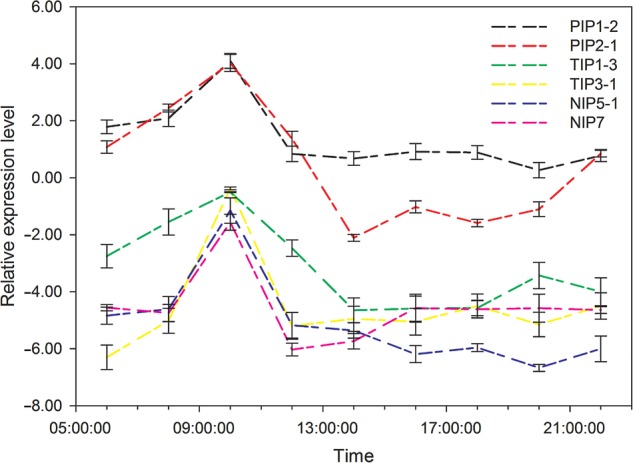
Diurnal expression of six AQPs in *T. ramosissima* from 6:00 to 22:00 on 25 June 2013.

**Figure 9.**
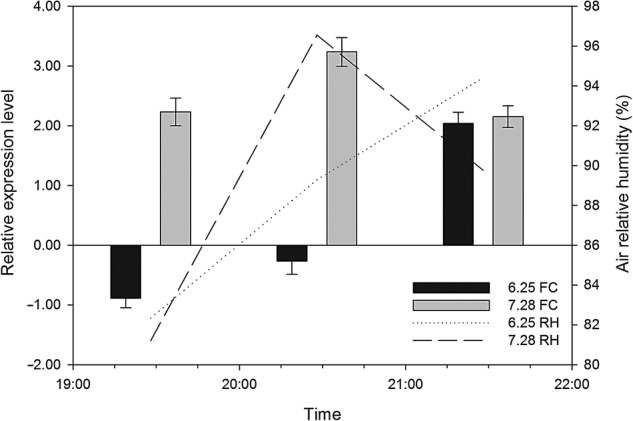
Comparison of the response of *PIP1-2* with different air moisture levels under natural conditions for 2 days. The black bars and dotted line show the expression level of *PIP2-1* and AirRh, respectively, on 25 June 2013. The grey bars and long dashed line show the expression level of *PIP2-1* and AirRh, respectively, for 28 June 2013.

A total of six AQP protein sequences were selected to construct a maximum likelihood phylogenetic tree using MEGA 6.0. The sequences were clustered into three main subgroups: PIPs (*PIP1-2* and *PIP2-1*), TIPs (*TIP1-1* and *TIP3-1*) and NIPs (*NIP5-1* and *NIP7*) (Fig. 7). Diurnal expressions of the six *AQP* genes, in the field under natural conditions, are shown in Fig. 8. The expression of the six *AQP*s was initiated rapidly at the beginning of sunrise (6:00) and reached a peak at 10:00. During this period, the expression levels of the *AQP*s increased with the intensity of PAR, which induced an increase in sap flow velocity. During midday, due to the shortage of a water supply, the expression of *AQP*s was sharply decreased to a minimum value at noon (12:30–13:30), and was maintained at a low value until 20:00. However, with the increase in AirRh after 20:00, the transcript levels of *PIP1-2*, *PIP2-1*, *TIP3-1* and *NIP5-1* increased again, though the amounts of the transcripts were weaker compared with those observed during the day. Compared with the lowest transcript level at 20:00, only *PIP2-1* was significantly upregulated (3.86-fold). Accordingly, the sap flow velocity was negative during the 20:00–22:00 time period, which suggested that *PIP2-1* was involved in foliar water uptake. The results of Pearson's correlation analyses also demonstrated that *PIP2-1* was the only gene that correlated tightly with AirRh (Table 3, *r*= −0.997, *P*-value = 0.048). To further validate the contribution of *PIP2-1* to foliar water uptake, another field experiment was performed in 28 July. There was an inflection point in the increase of AirRh, which was different in 25 June. Interestingly, the expression of *PIP2-1* increased with the inflection point (Fig. 9). We can, therefore, conclude that the *PIP2-1* gene is the major gene involved in the process of foliar water uptake.

**Table 3.** Pearson correlation coefficient between meteorological factors, AQP expression level and sap flow velocity from time 18 to 22. *Correlation is significant at the 0.05 level (two tailed). **Correlation is significant at the 0.01 level (two tailed). PCC, Pearson correlation coefficient; Sig._2, two-tailed test.

	AirRh	MS	S20	S10	MR	R20	PIP21	NIP7
AirRh								
PCC	1	−0.948	−0.995	−0.983	−0.882	−0.912	0.997*	0.978
Sig._2		0.206	0.066	0.118	0.313	0.269	0.048	0.133
MS								
PCC	−0.948	1	0.976	0.991	0.986	0.995	−0.921	−0.861
Sig._2	0.206		0.14	0.088	0.107	0.063	0.254	0.339
S20								
PCC	−0.995	0.976	1	0.997	0.926	0.95	−0.984	−0.951
Sig._2	0.066	0.14		0.052	0.247	0.203	0.115	0.199
S10								
PCC	−0.983	0.991	0.997	1	0.954	0.972	−0.966	−0.923
Sig._2	0.118	0.088	0.052		0.195	0.151	0.167	0.251
MR								
PCC	−0.882	0.986	0.926	0.954	1	0.998*	−0.843	−0.765
Sig._2	0.313	0.107	0.247	0.195		0.044	0.361	0.446
R20								
PCC	−0.912	0.995	0.95	0.972	0.998*	1	−0.878	−0.807
Sig._2	0.269	0.063	0.203	0.151	0.044		0.318	0.402
PIP21								
PCC	0.997*	−0.921	−0.984	−0.966	−0.843	−0.878	1	0.991
Sig._2	0.048	0.254	0.115	0.167	0.361	0.318		0.085
NIP7								
PCC	0.978	−0.861	−0.951	−0.923	−0.765	−0.807	0.991	1
Sig._2	0.133	0.339	0.199	0.251	0.446	0.402	0.085

### Effects of root pressure and high humidity on *PIP2-1* expression

**Figure 10.**
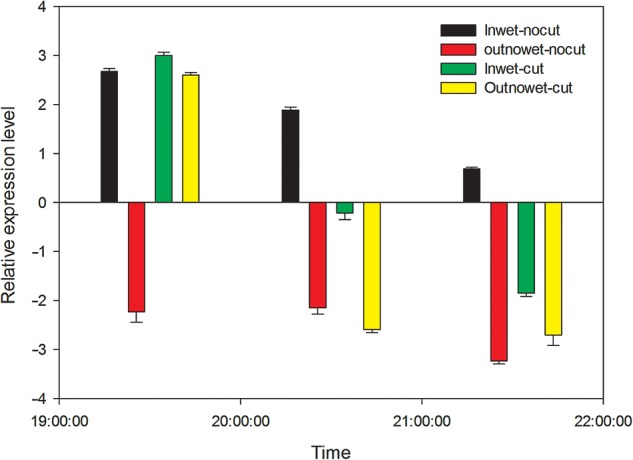
Impact of air moisture and root pressure on expression level changes in *PIP2-1* in natural field and control experiments. The humidity inside the chamber was over 85 %, and it was ∼50 % outside the chamber.

During days with high transpiration conditions, it is generally accepted that hydrostatic forces drive the radial flow of water across roots, and the apoplastic pathway is thought to be predominant. At night, when transpiration is low or null, water flow occurs via the water potential gradient built up by solute accumulation. To determine the effects of the root water pressure regulating *PIP2-1* expression at night, ‘cut’ and ‘nocut’ experiments with high air humidity exposure were performed using *T. ramosissima* (Fig. 10). At 19:30, in the presence of root water pressure under low AirRh conditions, the transcription level of *PIP2-1* remained low under natural conditions (Fig. 10, red bars), while it was upregulated under high AirRh conditions (black bars). When the presence of root water pressure was excluded under natural conditions (Fig. 10, yellow bar), the expression level of this gene was immediately upregulated to a level close to that observed under high air humidity exposure in natural conditions, and was even higher when the branches were exposed to high air humidity conditions (green bars). This phenomenon indicated that the *PIP2-1* gene is regulated by both air moisture and root water pressure at night. However, over time, the expression level of *PIP2-1* dropped in the presence or absence of root pressure, which suggested that the foliar water uptake regulated by *PIP2-1* is a short-term response process. Compared with the expression level of *PIP2-1* in the presence of root water pressure, its duration was shorter, suggesting that foliar water uptake is a subsidiary strategy for desert plants in order to adapt to extreme drought, and might not be sufficient alone for sustaining the growth of plants.

## Discussion

Previous studies have reported that the natural distribution of *Tamarix* plants is correlated with the depth and stream of underground water (Zhuang and Chen 2006; Li *et al*. 2013), which suggests that underground water, collected through the roots, is the major resource for this desert plant (Anderson 1982; Zhao *et al*. 2008; Liu and Zhao 2009). Even under conditions of high air moisture, foliar water uptake has a negligible impact on water hydraulic adhesion in plants when abundant water is supplied from the roots, such as under conditions with abundant precipitation or well-irrigated environments. However, foliar water uptake cannot be ignored in some extreme cases. In our present experimental field, since transplantation in the year 2003, *T. ramosissima* plants had not been watered or irrigated, nor was the field close to irrigated land. Moreover, in this area, there was also little precipitation since transplantation, particularly in the year 2013 when the annual precipitation was not >90 mm. Due to the scarcity of root water input, the foliar absorption phenomenon was more likely to occur.

A previous investigation of the water content of the different leaves of *T. ramosissima* plants showed that the leaf water content significantly increased by the end of submergence (Li *et al*. 2014). Initially, before moisture is added, tender leaves have slightly higher water content than mature leaves. However, when moisture is added, mature leaves have a slightly higher water content than tender leaves. Ultimately, the water content increased by 29.38 % in the mature leaves, while it increased by 20.93 % in the tender leaves (absorption: ∼0.462 g g^−1^ for mature leaves and 0.341 g g^−1^ for tender leaves; **see Supporting Information—Fig. S5**). From 20:00 to 22:00 in 25 June, considering that the AirRh increased from 38.89 to 59.04 % and that the diurnal water content of the soil did not show much variation (3.79–3.802 %), the increase in water content of leaves from 65.75 to 67.45 % (Fig. 4) can be mainly attributed to the AirRh. Interestingly, during these periods of air moisture absorption, there was a significant gradient in water potential from leaves to stems (Fig. 6), which induced sap flow velocity in the negative direction. These phenomena were aggregated under higher humidity exposure conditions **[see Supporting Information—Fig. S6]**. As shown in **Supporting Information—Fig. S7**, under high humidity exposure to mineral water (low δ^18^O content), isotopic δ^18^O content in leaves decreased immediately, but decreased slightly in stems, which suggested that leaves not only absorbed water but also somehow stored water.

During the daytime, sap flow velocity, relative conductivity and water potential of leaves of *T. ramosissima* display a similar pattern to that of other species, suggesting that the hydraulic adhesion of this desert plant is largely contributed by light-dependent transpiration and root pressure, similar to other plants (Henzler *et al*. 1999; Moshelion *et al*. 2002; Lopez *et al*. 2003; Hachez *et al*. 2008; Sakurai-Ishikawa *et al*. 2011; Šurbanovski *et al*. 2013). As major channels for controlling the water content in plants, AQPs can mediate transcellular water flux between different parenchymal cells and between parenchymal cells and xylem vessels (Maggio and De Pascale 2007). Most of the expression of *AQP* genes is light-dependently regulated by transpiration (Henzler *et al*. 1999; Moshelion *et al*. 2002; Lopez *et al*. 2003; Beaudette *et al*. 2007; Ohrui *et al*. 2007; Hachez *et al*. 2008). In our study, we also found that the expression of the six *AQPs* increased from 8:00 to 10:00 with enhanced PAR (Fig. 8). However, with increasingly intense sunshine, their expression was sharply depressed, which is in agreement with a previous study that showed that *AQP* expression was more favoured by shade than darkness and full sunlight (Voicu *et al*. 2009). In contrast, we noticed that a second peak in *AQP* expression occurred after 16:00, which is consistent with other studies (Lopez *et al*. 2003; Šurbanovski *et al*. 2013). They attributed this phenomenon to post-transcriptional regulation, although there was a different humidity level examined during the day (60 %) and at night (85 %) in their study. In our study, *T. ramosissima* foliar water uptake could have been initiated by exposure to high humidity (over 80 %; Li *et al*. 2014). We found that the *PIP2-1* gene was significantly induced by an increase in AirRh at night (Figs 8 and 9), which suggested that the expression of the *PIP2-1* gene may be independent of light and transpiration. Further experiments to measure the protein levels need to be conducted to verify the regulation network of this *AQP* gene.

In addition, there are over 30 *AQP* gene isoforms in the genomes of model plants, although the exact number varies (Chaumont *et al*. 2001; Sakurai *et al*. 2005). The number of *AQP* gene copies in the genome of *T. ramosissima* remains unknown. In this study, the primers used for RT-PCR were based on the sequences of each gene from the *de novo* transcriptome of *T. hispida*, because we do not have sequence information of AQPs from *T. ramosissima*. We recently obtained the *de novo* transcriptome of *T. ramosissima*, which facilitated comparisons of the homology between the two *Tamarix* species. Although many SNPs were identified between the two species, the primer worked well due to the position on the conserved region of the sequences **[see** **Supporting Information—Fig. S8****]**. Sequence alignments of *PIP1-2* and *PIP2-1* were also performed (identity was 0.59), and both forward and reverse primers were totally distinct from each other. There may be genes in addition to the *AQP*s involved in the process of foliar water uptake. We believe that with more knowledge of the transcriptome and genome of *T. ramosissima*, the roles, functional diversification and expression regulation of all *AQP* genes could be clarified in future studies.

## Conclusions

In extreme drought environments in desert regions, soil water content is low because of scarce rainfall. Plants cannot sustain their growth and reproduction using water from roots alone, and other strategies such as foliar water uptake may be an important supply for desert plants to alleviate drought stress. In this study, we show that when the AirRh increases at night, the water content of leaves increases, resulting in a water potential gradient between SPAC, which drives water transport from the air to leaves and stores it in the stems of *T. ramosissima*. As major channels for controlling water content in plants, AQPs were selected to determine their contribution to foliar water uptake. During the day, AQP regulation process did not differ from the traditional light-mediated process using transpiration. In contrast, at night, the *PIP2-1* gene was induced by the increasing AirRh, which is independent of light and transpiration. We found that the *PIP2-1* gene may be one of the *AQP* genes responsible for foliar water uptake in *T. ramosissima*. To the best of our knowledge, this study is the first to combine field and control experiments to examine foliar water uptake, and is also the first to elucidate the molecular mechanisms of leaf water absorption in desert plants.

## Sources of Funding

This work was funded by the ‘One Hundred Talents’ Project of the Chinese Academy of Sciences (grant no. 29Y127E71) and National Natural Science Foundation of China (nos 91125025 and 31500266).

## Contributions by the Authors

X.Y., H.X. and X.-F.M conceived and designed the experiments; X.Y., X.D. and M.Z performed the experiments; X.Y., X.D., S.Z. and X.-F.M. analysed the data; X.-F.M. contributed reagents/materials/analysis tools and X.Y., X.D. and X.-F.M. wrote the paper.

## Conflict of Interest Statement

None declared.

## Supporting Information

The following additional information is available in the online version of this article –

**File S1.** Diurnal soil water content variation on 25 June 2013.

**Figure S1.** Soil water potential in Sitan.

**Figure S2.** Humidity variation from June to September 2013.

**Figure S3.** Protein sequences used for phylogenetic tree reconstruction.

**Figure S4.** Seven figures of melting curves for the reference gene GAPDH and the six aquaporins examined in RT-PCR experiments.

**Figure S5.** Water contents of *T. ramosissima* leaves and shoots for the control and treatment groups.

**Figure S6.** Water potential difference and relative humidity at specific times over 3 days.

**Figure S7.** Comparison of isotope values in leaves and stem branches under high humidity.

**Figure S8.** Comparison of homologous aquaporins between *T. ramosissima* and *T. hispida*.

Additional Information
