# Supplementary material for: Molecular mechanisms of foliar water uptake in a desert tree
Source: AoB Plants. 2015 Nov 13;7:plv129. doi: 10.1093/aobpla/plv129 (PMC4685171; doi:10.1093/aobpla/plv129)
Supplement: Additional Information [file supp_7_plv129_index.html]

Molecular mechanisms of foliar water uptake in a desert tree — Molecular mechanisms of foliar water uptake in a desert tree — Additional Information 

# Molecular mechanisms of foliar water uptake in a desert tree

## Additional Information

Additional Information

- Additional Information - Docx file
- Supplementary file1 - jpg file
- Supplementary file2 - tif file
- Supplementary file3 - jpg file
- Supplementary file4 - txt file
- Supplementary file5 - tif file
- Supplementary file6 - jpg file
- Supplementary file7 - tif file
- Supplementary file8 - tif file
