# Supplementary material for: Molecular mechanisms of foliar water uptake in a desert tree
Source: AoB Plants. 2015 Nov 13;7:plv129. doi: 10.1093/aobpla/plv129 (PMC4685171; doi:10.1093/aobpla/plv129)
Supplement: Additional Information [file supp_plv129_plv129supp.docx]

**File S1** Diurnal soil water content variation on 25^th^ June 2013.

**File S2** Soil water potential in Sitan.

**File S3** Humidity variation from June to September 2013.

**File S4** Protein sequences used for phylogenetic tree reconstruction

**File S5** Seven figures of melting curves for the reference gene GAPDH and the 6 aquaporins examined in RT-PCR experiments.

**File S6** Water contents of *T. ramosissima* leaves and shoots for the control and treatment groups

**File S7** Water potential difference and relative humidity at specific times over three days.

**File S8** Comparison of isotope values in leaves and stem branches under high humidity

**File S9** Comparison of homologous aquaporins between *T .ramosissima* and *T. hispida.*
