# Supplementary figures and images for: Molecular mechanisms of foliar water uptake in a desert tree
Source: AoB Plants. 2015 Nov 13;7:plv129. doi: 10.1093/aobpla/plv129 (PMC4685171; doi:10.1093/aobpla/plv129)

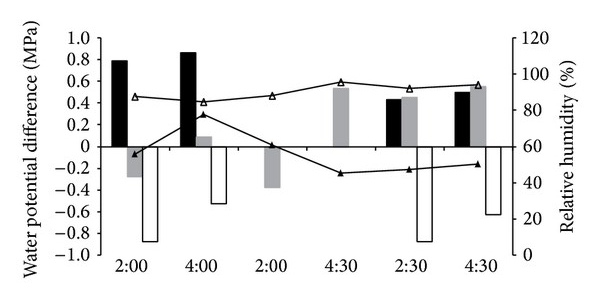

Supplement: Additional Information [file supp_plv129_plv129supp_file1.jpg]

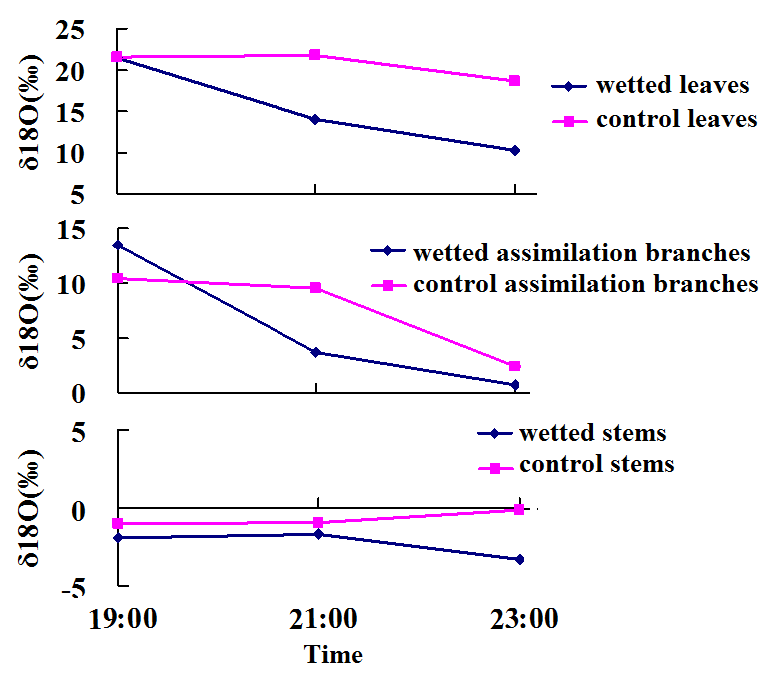

Supplement: Additional Information [file supp_plv129_plv129supp_file2.tif]

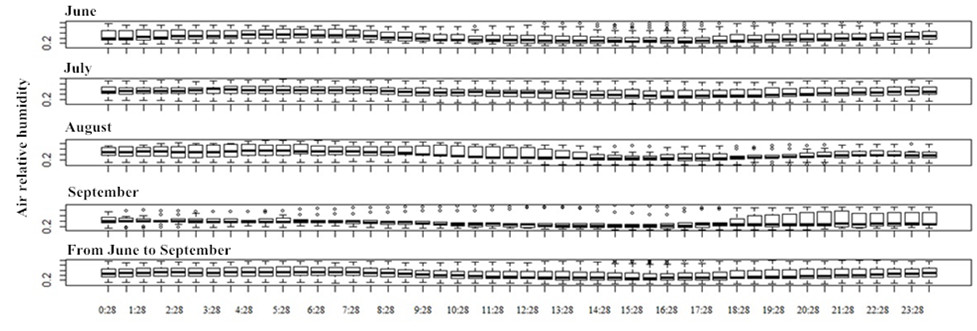

Supplement: Additional Information [file supp_plv129_plv129supp_file3.jpg]

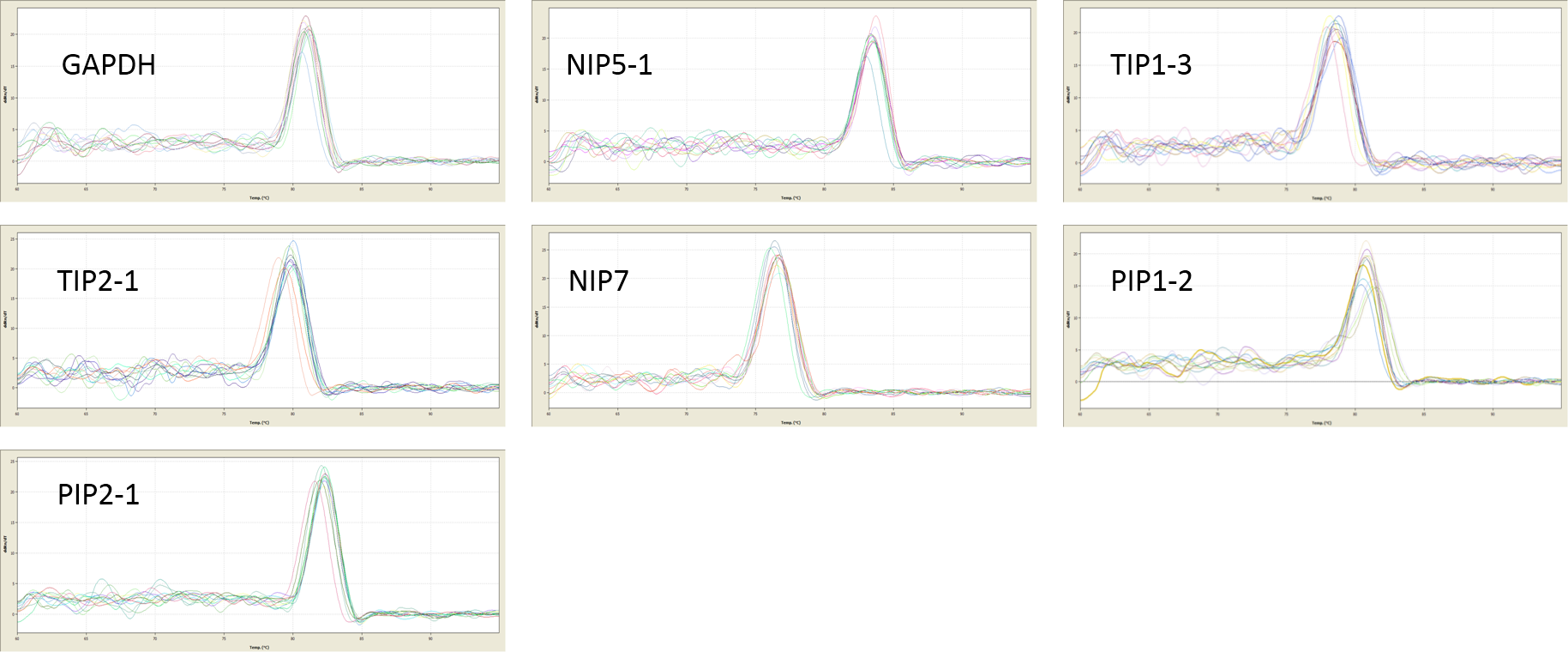

Supplement: Additional Information [file supp_plv129_plv129supp_file5.tif]

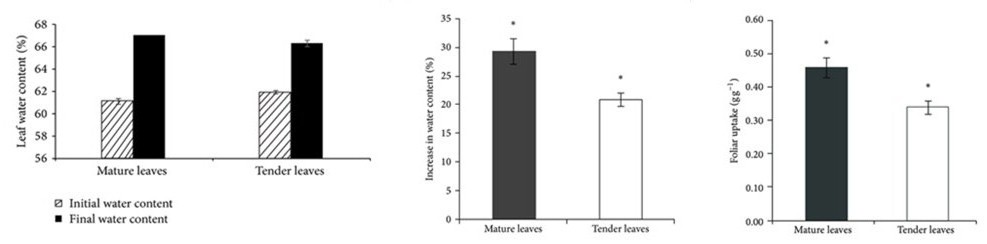

Supplement: Additional Information [file supp_plv129_plv129supp_file6.jpg]

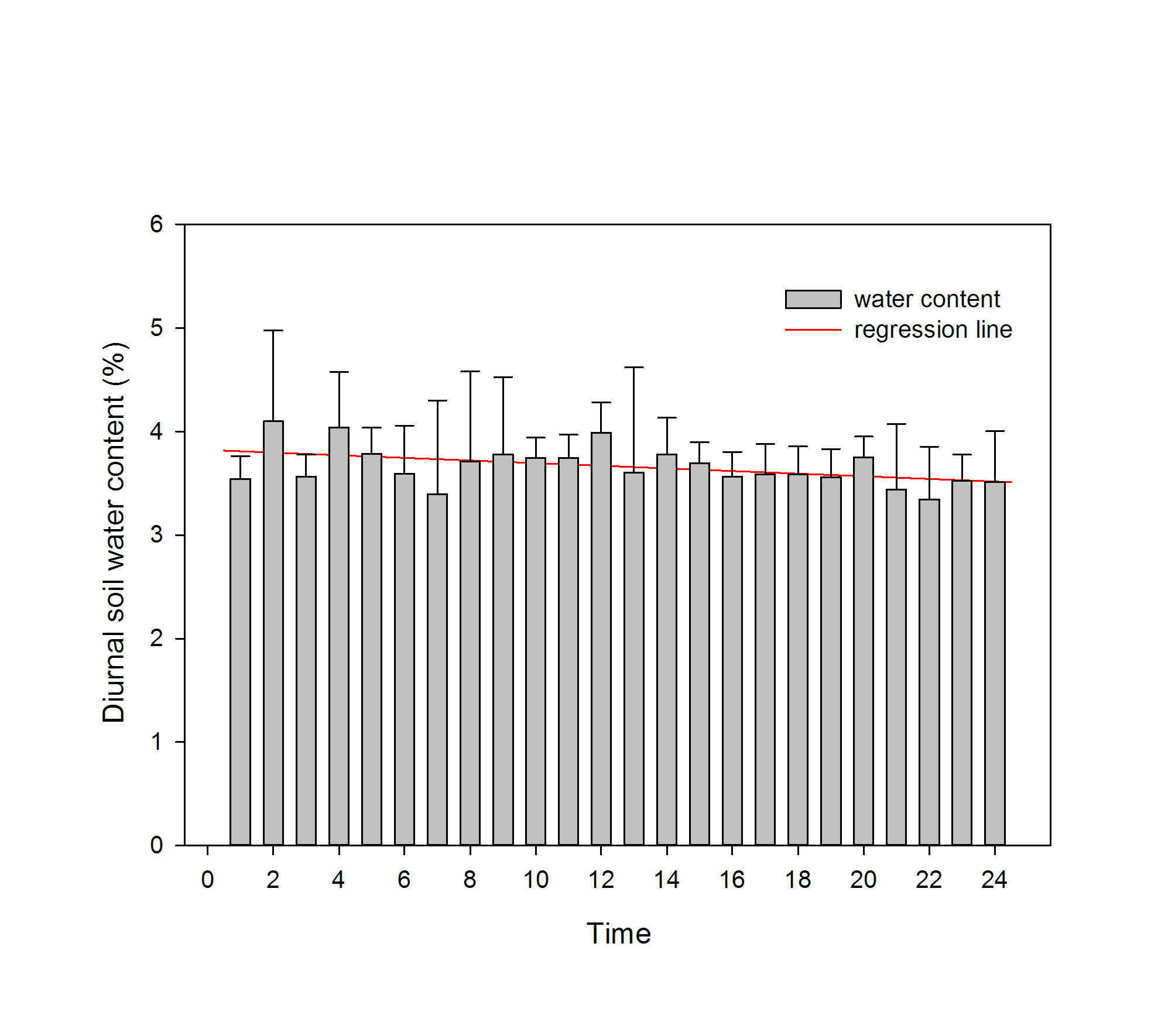

Supplement: Additional Information [file supp_plv129_plv129supp_file7.tif]

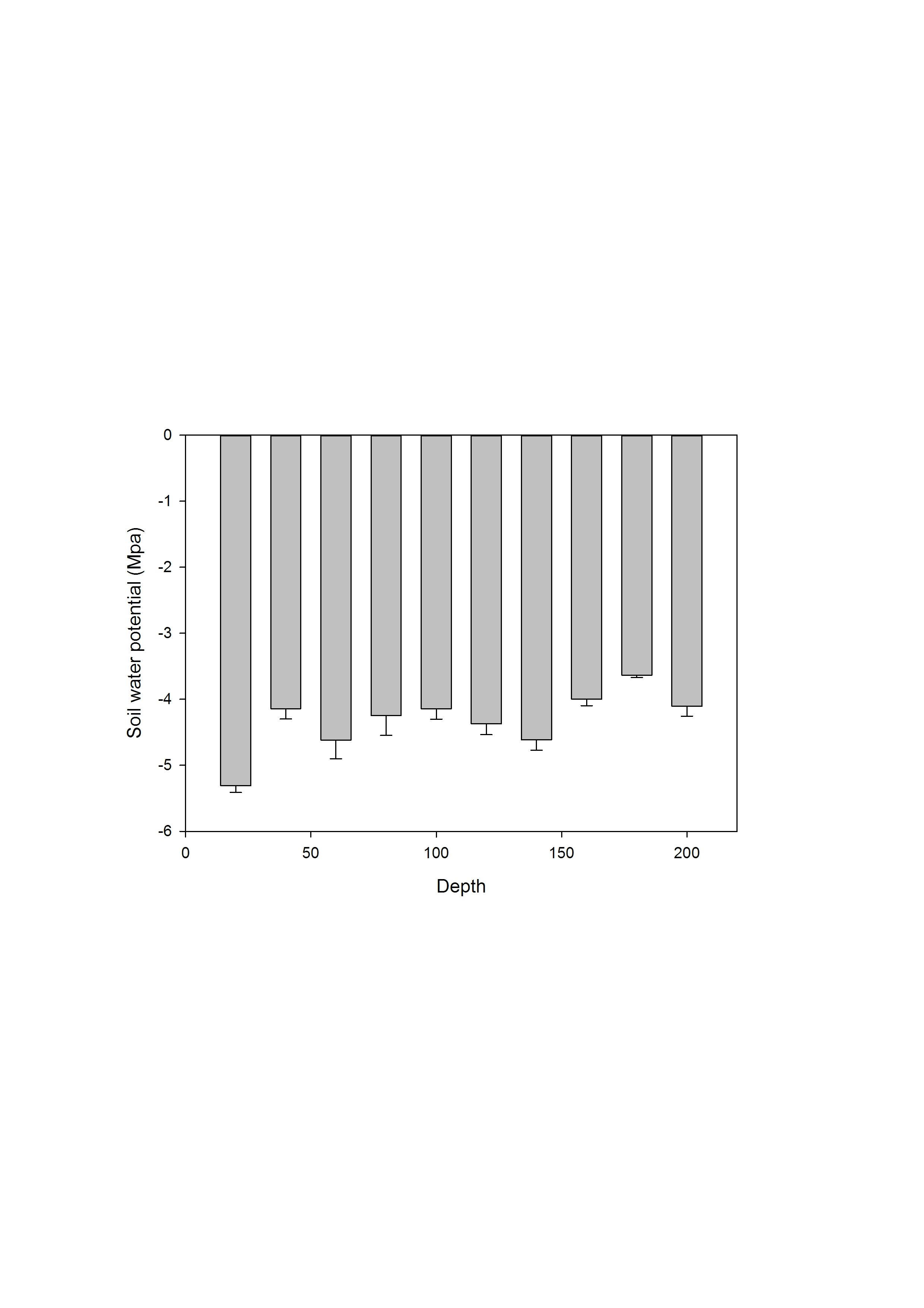

Supplement: Additional Information [file supp_plv129_plv129supp_file8.tif]
